# Vitamin D Knowledge, Attitudes, Practices and Serum Concentration Among Pregnant Women Attending a Malaysian Tertiary Hospital

**DOI:** 10.1002/fsn3.70575

**Published:** 2025-07-09

**Authors:** Yakubu Ibrahim, Amilia Afzan Mohd Jamil, Nurul Iftida Basri, Simran Lau Sher Reen, Muhammad Daniel Iman Asmunni, Nirosha Govidarajoo

**Affiliations:** ^1^ Department of Obstetrics and Gynaecology, Faculty of Medicine and Health Sciences Universiti Putra Malaysia (UPM) Serdang Selangor Malaysia; ^2^ Department of Medical Laboratory Science, Faculty of Allied Health Sciences, College of Medical Sciences Ahmadu Bello University Zaria Kaduna State Nigeria

**Keywords:** attitudes, knowledge, practices, pregnant women, vitamin D, vitamin D deficiency

## Abstract

Vitamin D deficiency is increasingly recognized as a public health concern among pregnant women due to its association with adverse pregnancy outcomes. Inadequate knowledge, attitudes, and practices (KAP) related to vitamin D may contribute to this issue. The study aimed to evaluate the KAP and serum vitamin D concentration among pregnant women. The study was a hospital‐based cross‐sectional study involving pregnant women residing in the Klang Valley area. Relevant data on sociodemographic characteristics, knowledge, attitudes, practices, and physical activity were collected using a structured questionnaire. Blood samples were collected using the veni puncture technique, and serum vitamin D levels were measured using the electrochemiluminescence immunoassay (ELCIA) technique. There was a significantly higher proportion of participants with good knowledge as compared with participants with poor knowledge (54.1% vs. 45.9%); *p* = 0.015. There was a significantly higher proportion of participants with negative attitudes as compared with positive attitudes (54.8% vs. 45.2%); *p* = 0.0001 and inconsistent practice level (50.7% vs. 49.3%); *p* = 0.953. The prevalence of vitamin D deficiency was 64.4%. Further analysis revealed that non‐Malay participants had significantly higher odds of having good knowledge of vitamin D compared to Malays (aOR = 0.420, *p* = 0.027). Employment status was significantly associated with good practices. Employed participants were two times more likely to demonstrate vitamin D‐related practices (aOR = 2.036, *p* = 0.049) compared to their unemployed counterparts. There were no significant associations between sociodemographic characteristics and participants' attitudes on vitamin D. Despite the high levels of knowledge about vitamin D among the participants, the majority had negative attitudes, inconsistent practice levels, and a high prevalence of vitamin D deficiency. The findings underscore the need for policies and culturally acceptable strategies aimed at promoting attitudes and practices to prevent vitamin D deficiency in pregnancy.

## Graphical abstract

This hospital‐based cross‐sectional study assessed knowledge, attitudes, and practices (KAP), and serum vitamin D concentration among pregnant women in Klang Valley, Malaysia. The majority of the participants demonstrated good knowledge, negative attitudes, inconsistent practices, and a high prevalence of vitamin D deficiency. Findings highlight the need for policies and culturally acceptable strategies to address vitamin D deficiency in pregnancy.

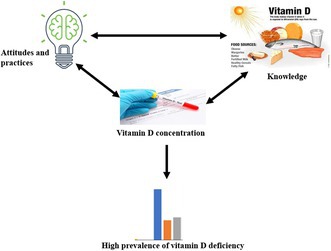

## 1 Introduction

Vitamin D deficiency is increasingly recognized as a significant non‐communicable public health concern across many developing countries, including those in Asia, Africa, the Middle East, and Latin America, despite their abundant sunlight exposure (Bonevski et al. 2013; Van Schoor and Lips 2011). Vitamin D, a steroid hormone essential for calcium absorption and bone health, exists in two major forms: vitamin D_2_ (ergocalciferol), derived from plant sources, and vitamin D_3_ (cholecalciferol), synthesized in the skin upon exposure to ultraviolet B (UVB) radiation and also obtained from animal‐based foods (Davoudi‐Kiakalayeh et al. 2017). Dietary sources of vitamin D include oily fish such as salmon, mackerel, sardines, and tuna; egg yolks; fortified dairy products such as milk, cheese, and yogurt; and supplements (Bonevski et al. 2013; Van Schoor and Lips 2011).

In many low and middle‐income countries (LMICs), particularly among pregnant women, vitamin D deficiency is alarmingly widespread (Roth et al. 2018). Several interrelated factors contribute to this problem, including limited sun exposure due to cultural and religious clothing practices, urbanized indoor lifestyles, and high levels of air pollution, all of which reduce cutaneous vitamin D synthesis (Lee et al. 2021; Prentice et al. 2009). In addition, low dietary intake of vitamin D‐rich foods and minimal use of supplementation during pregnancy further compound the issue (Lee et al. 2021; Spiro and Buttriss 2014). Vitamin D deficiency is prevalent among pregnant women globally, especially in low‐resource settings (Roth et al. 2018; Saraf et al. 2016) and lack of health education and awareness is a key barrier to improving vitamin D levels in pregnancy (Burchell et al. 2020; Palacios and Gonzalez 2014).

Vitamin D deficiency in pregnancy has been associated with a range of adverse maternal and neonatal outcomes (Dovnik and Mujezinović 2018), including gestational diabetes mellitus (GDM) (Alzaim and Wood 2013; Colonese et al. 2015; Liu 2021), hypertensive disorders of pregnancy (Achkar et al. 2015; Bodnar et al. 2014; Burris et al. 2014), preterm birth (Kalok et al. 2020; Wei 2014) and fetal growth restriction (Escudero et al. 2016), which significantly contribute to maternal and neonatal morbidity and mortality globally.

Prior research across populations around the globe has linked low vitamin D status to risk factors that affect vitamin D levels, for example, dietary habits and cultural and religious teachings on wearing dark veils covering the entire body parts thereby preventing sunlight exposure (Escudero et al. 2016; Žmitek et al. 2021). Moreover, there are no laws governing the fortification of vitamin D in foods in many countries (Spiro and Buttriss 2014).

Despite Malaysia's geographic location near the equator and its consistent year‐round sunlight, vitamin D deficiency remains a significant concern among Malaysian pregnant women, primarily driven by cultural norms, clothing practices, sun‐avoidance behavior, urban lifestyle, and limited dietary intake of vitamin D‐rich foods (Bukhary et al. 2016; Chee et al. 2022; Lee et al. 2021; Mohamed et al. 2014; Woon et al. 2019). Perhaps, the daily median total vitamin D intake is below the Institute of Medicine (IOM) and recommended nutrient intake (RNI) (15 μg/day) for Malaysian pregnant women (IOM 2011; MOH Malaysia 2017). Several studies conducted in Klang Valley, a densely populated and urbanized region in Malaysia, have reported alarmingly higher rates of vitamin D deficiency among pregnant women, with deficiency rates ranging from 42.6% to 90.4% during the second and third trimesters of gestation (Akma et al. 2021; Bukhary et al. 2016; Isa et al. 2022; Jamil et al. 2019; Lee et al. 2021; Palaniveloo et al. 2020; Woon et al. 2019). Despite these alarming numbers, few studies have investigated the knowledge, attitudes, and practices (KAP) in relation to vitamin D concentration in pregnant women. Evaluation of KAP alongside biochemical assessment of vitamin D levels is essential for informing policies and formulating effective health awareness programs. The study aimed to assess the knowledge, attitudes, practices, and serum vitamin D concentration among pregnant women attending a tertiary hospital in Klang Valley, Malaysia.

## 2 Materials and Methods

### 2.1 Study Design

This cross‐sectional study was conducted between May 2022 and February 2023. A validated, English‐language questionnaire was used for the data collection. A total of 146 pregnant women residing in Klang Valley and attending the antenatal clinic in Hospital Sultan Abdul Aziz Shah (HSAAS) were recruited into the study using a convenience sampling method.

### 2.2 Inclusion and Exclusion Criteria

**FIGURE 1:**
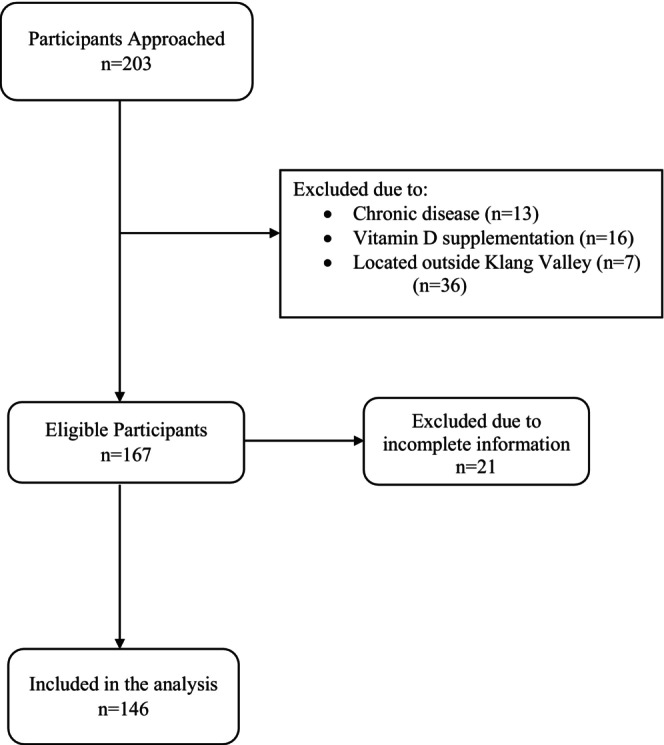
Participants recruitment flowchart.

Pregnant women residing in the Klang Valley area were recruited into the study. Pregnant women taking vitamin D supplements or any medication that could interfere with vitamin D metabolism, participants with chronic diseases, or those not residing in the Klang Valley area were excluded from the study. A total of 203 participants were targeted to participate in the study. Of these, 36 were excluded based on the study's exclusion criteria: 13 had chronic medical conditions such as diabetes or renal disease, 16 were taking vitamin D supplements, and seven participants were not residing in the Klang Valley area. This resulted in 167 participants who met the eligibility criteria. However, 21 of the eligible participants had incomplete information. Consequently, 146 participants were included in the analysis. The participant's recruitment flowchart was shown in Figure 1.

### 2.3 Instrument Validity and Reliability

The questionnaire used in this study was adapted and modified from a previous study (Jamil et al. 2019). It comprised two main sections: Part I collected sociodemographic information, while Part II was further divided into four parts: I, II, III, and IV.

Part I consisted of 12 statements assessing participants' knowledge level on vitamin D. Each question had three response options: “Correct,” “Wrong,” or “Not sure.” Correct responses were awarded 2 points, while incorrect and “Not sure” responses received 0 and 1 point, respectively. The total knowledge score ranged from 0 to 24. Median knowledge scores were calculated, and participants were classified as having either poor (below the median) or good (above the median) knowledge. Knowledge responses were presented in Table S1.

Part III assessed the attitude levels related to vitamin D, using 12 statements rated on a 5‐point Likert scale: “Strongly disagree,” “Disagree,” “Neutral,” “Agree,” and “Strongly agree.” Scoring used was; 0 point for “Strongly disagree”/“Disagree,” 1 point for “Neutral,” 2 points for “Agree,” and 3 points for “Strongly agree.” The total attitudes score was compared against the median to classify attitudes as either negative (below the median) or positive (above the median). Attitude responses were shown in Table S2.

Part III assessed participants' practice levels related to vitamin D during pregnancy using 12 items rated on a 5‐point Likert scale: “Never,” “Rarely,” “Sometimes,” “Often,” and “Always.” Scoring for positive practices was as follows: 0 points for “Never,” 1 point for “Rarely,” 2 points for “Sometimes,” 3 points for “Often,” and 4 points for “Always.” Total practice score ranges from 1 to 42. The median score was calculated, and participants were categorized as having poor (below the median) or good (above the median) practice. Practice‐related responses were presented in Table S3.

Part IV of the questionnaire assessed the participants' physical activity. The Pregnancy Physical Activity Questionnaire (PPAQ) was adapted from previously published guidelines (Chasan‐taber et al. 2004; Syed Nor et al. 2022). To estimate daily energy expenditure and classify participants according to metabolic equivalent task (MET) levels, responses were summed to generate a total score. This total was divided by the number of questions and then by 7 (days of the week) to obtain the average daily METs. Participants were then categorized as follows: sedentary (< 1.5 METs), light activity (1.5 to < 3.0 METs), moderate activity (3.0–6.0 METs), or vigorous activity (> 6.0 METs) (Connor et al. 2018). The physical activity responses were shown in Table S4.

Furthermore, we performed a sensitivity analysis to determine the validity of KAP categorization using median scores. Besides using the median as the main cut‐off point, we also tested other methods, such as using the mean score and dividing the scores into quartiles. In addition to using the median as the primary cut‐off point, using these various categories, we then re‐examined the key findings, ensuring the results remained consistent, indicating the reliability of using the median method for categorizing the KAP scores.

To ensure the validity and reliability of the questionnaire, a multi‐step process was carried out, including expert review, pretesting, and statistical reliability analysis. The initial draft of the questionnaire was reviewed by a panel of three experts, including two nutritionist professionals with expertise in maternal health nutrition and one academic with experience in questionnaire design. The experts evaluated the items for relevance, clarity, and alignment with the study objectives. Based on their feedback, several questions were refined to improve clarity and ensure that the items appropriately captured the constructs of knowledge, attitudes, and practices related to vitamin D among pregnant women.

Furthermore, the revised questionnaire was pretested with 14 pregnant women (approximately 10% of the calculated sample size) who were not part of the final study. The pretest aimed to assess the comprehensibility, length, and structure of the questionnaire based on the participants' perspective. Participants were asked to provide feedback on any items that were unclear or difficult to understand. Minor adjustments were made based on this feedback. After finalizing the questionnaire, the internal consistency was assessed using Cronbach's alpha. A Cronbach's alpha value of ≥ 0.70 was considered acceptable for indicating good internal consistency. Items that did not meet this value were reviewed for potential ambiguity or redundancy and were either revised or excluded from the final questionnaire. Participants involved in the pretesting and validation process were subsequently excluded from the main study to avoid any potential bias in responses.

### 2.4 Blood Sample Collection

Using a standard venipuncture technique, about 5.0 mL of blood was collected from the antecubital vein of each participant by a qualified phlebotomist and dispensed into a plain vacutainer tube (BD Scientific, United Kingdom). Samples were transported to the laboratory under cold chain. The samples were centrifuged at 4000 rpm for 10 min. The clear, non‐hemolyzed serum was then separated into two aliquots in a pyrogen‐free tubes (MDHC Life Technologies, China) and stored at −20°C until analysis.

### 2.5 Laboratory Analytical Protocol for Vitamin D

Serum vitamin D levels were analyzed using the Electrochemiluminescence Immunoassay (ECLIA) technique (Roche, Germany). Specifically, the metabolite of vitamin D, 25‐hydroxycholecalciferol (25(OH)D_3_) was used as a marker for the assessment of vitamin D (Holick et al. 2007).

Quality control was ensured by including per run control sera representing deficient, insufficient, and normal vitamin D levels. All laboratory procedures strictly followed standard quality control protocols. Vitamin D status was categorized based on the IOM's guidelines for diagnosing vitamin D status: deficient (< 30 nmol/L), insufficient (30–50 nmol/L), and normal (> 50 nmol/L) (Connor et al. 2018).

### 2.6 Statistical Analysis of Data

Data were analyzed using the IBM SPSS Statistics software, version 27.0 (Armonk, NY, USA). Descriptive statistics were used to summarize frequencies, proportions, medians, minimum, and maximum values for KAP scores, as well as serum 25(OH)D_3_ concentrations. Mann—Whitney *U*‐test was used to determine the significant difference of median between KAP scores and KAP outcome levels. Association between sociodemographic characteristics and KAP was analyzed using Chi square analysis and a binary logistic regression model. Statistical significance was determined at *p* ≤ 0.05 at a 95% confidence interval (CI).

## 3 Results

### 3.1 Socio‐Demographic Information of the Study Subjects

The majority of participants were between 21 and 27 years (42.7%), and predominantly of Malay ethnicity (69.2%), and had attained tertiary education (69.9%). Most participants reported a middle monthly household income (42.0%), and over half were nulliparous (54.5%) and in their third trimester of pregnancy (73.3%). Regarding physical activity, a large proportion of the participants (83.2%) were classified as sedentary (< 1.5 MET). In terms of vitamin D status, 64.4% of participants were vitamin D deficient (< 30 nmol/L), 29.5% were insufficient (30–49 nmol/L), and only 6.2% had normal levels (≥ 50 nmol/L), as shown in Table 1.

**TABLE 1:** Sociodemographic characteristics, physical activity levels, and serum 25(OH)D concentration among the study participants.

Variables	Characteristics	Frequency (*N*)	Percentage
Age (years)	≤ 20	5.0	3.4
	21–27	62.0	42.5
	28–34	43.0	29.5
	≥ 35	36.0	24.7
Education level	Secondary and above	137	93.8
	Below secondary	9.0	6.2
Ethnicity	Malay	100.0	68.5
	Chinese	22.0	15.1
	Indian	21.0	14.4
	Others	3.0	2.1
Household income	Low (B40)	56	38.4
	Middle (M40)	61	41.8
	High (T20)	29	19.9
Gestation age	1–12 weeks	3	2.1
	> 12 weeks	143	97.9
Parity	Nulliparous	79	54.1
	Multiparous	67	45.9
Physical activity level	Sedentary activity	121	82.9
	Light activity	19	13.0
	Moderate activity	6.0	4.1
	Vigorous activity	0.0	0.0
Vitamin D status	Deficient	94.0	64.4
	Insufficient	43.0	29.5
	Normal	9.0	6.2

*Note:* B40 is the bottom 40% income earners in the population; M40 is the middle 40% income earners, T20 is the top 20% income earners; Sedentary activity (< 1.5 MET); Light activity (1.5–3.0 MET); Moderate activity (3.0–6.0 MET); Vigorous activity (> 6.0 MET); vitamin D status; deficient, < 30 nmol/L, insufficient, 30–50 nmol/L, and normal, > 50 nmol/L.

### 3.2 Knowledge, Attitude, and Practice Among the Study Participants

Table 2 shows the classification and score of knowledge, attitudes, and practices among the study subjects. The knowledge score had a median (IQR) of 12 (8.0), with a maximum score of 24. Of the participants, 79 (54.1%) scored above the median knowledge score, and 67 (45.9%) scored below the median knowledge score. Notably, there was a statistically significant difference (*p* = 0.015) in the higher proportion of subjects having good knowledge compared to participants with poor knowledge.

**TABLE 2:** Scores and classification of knowledge, attitudes, and practices among the study participants.

Variable	Indices	Median (IQR)	Min	Max
Knowledge score		12 (8.0)	0	24
Attitudes score		20 (7.3)	2	36
Practices score		19 (12.0)	1	42
25(OH)D_3_ concentration (nmol/L)		33 (20.2)	14	95

		*N* (%)	*p*
Knowledge level	Poor	67 (45.9)	0.015^†,^*
	Good	79 (54.1)	
Attitudes level	Negative	80 (54.8)	0.0001^†,^*
	Positive	66 (45.2)	
Practices level	Bad	74 (50.7)	0.953^**†**^
	Good	72 (49.3)	

*Note:* Values were expressed as median (IQR), IQR is the interquartile range. The level of significance was considered at *p* ≤ 0.05; vitamin D status; deficient, < 30 nmol/L, insufficient, 30–50 nmol/L, and normal, > 50 nmol/L. ^**†**^Mann–Whitney *U*‐test significant. The level of significance and was considered at *p ≤ 0.05.

The total attitude score had a median (IQR) of 20 (7.3), with a maximum score of 36 points. Of the participants, 66 (45.2%) scored above the median attitude score, while 80 (54.8%) subjects scored below the median score. There was a significant (*p* = 0.001) higher proportion of participants with negative attitudes compared to those with positive attitudes.

The total practice score had a median (IQR) of 19 (12.0), with minimum and maximum scores of 2 and 42, respectively. Of the participants, 74 (50.7%) scored above the median practice score, while 72 (49.3%) scored below the median. There was no statistically significant difference in the proportion of participants with good practice and those with bad practice related to vitamin D.

### 3.3 Sociodemographic Characteristics, Physical Activity and Vitamin D Status Among the Study Subjects

About 42% of the participants were between 21 and 27 years, predominantly of Malay descent (68.5%). About 94% of participants had educational attainment above secondary education. Most participants were middle‐income earners (42.0%), and approximately 98% were at > 12 weeks of gestation; 54% were nulliparous and 46% were multiparous. Regarding physical activity, a large proportion of the participants (83%) were classified as having sedentary activity (< 1.5 MET). Additionally, our analysis of vitamin D status revealed that the majority of participants were vitamin D deficient (64.4%), insufficient (29.5%) and only 6.2% of participants had normal levels, as shown in Table 1.

### 3.4 Knowledge, Attitude, and Practice Among the Study Participants

Table 2 shows the classification and score of knowledge, attitudes, and practices among the study participants. The knowledge score had a median (IQR) of 12 (8.0), with a maximum score of 24. Of the participants, 36.9% scored above the median knowledge score and 31.3% scored below the median knowledge score. Notably, there was a significantly (*p* = 0.015) higher proportion of subjects with good knowledge compared to participants with poor knowledge.

The median (IQR) of attitudes score was 20 (7.3), with a maximum score of 36 points. Of the participants, 30.8% scored above the median attitudes score, while 37.4% scored below the median score. There was a significantly (*p* = 0.001) higher proportion of participants with negative attitudes compared to those with positive attitudes.

The total practice score had a median (IQR) of 19 (12.0), with minimum and maximum scores of 1 and 42, respectively. Of the participants, 34.6% scored above the median practice score, while 33.6% scored below the median. There was no significant difference in the proportion of participants regarding the practices related to vitamin D.

### 3.5 Vitamin D Knowledge, Attitudes, Practices, and Sociodemographic Characteristics Among the Study Participants

Table 3 showed the associations between sociodemographic characteristics, physical activity levels, and knowledge, attitudes, and practices levels among the participants. Our analysis shows that non‐Malay participants demonstrated a higher proportion of good knowledge (66.0%) compared to Malays (46.9%). There was a significant association between ethnicity and knowledge level (*χ* ^2^ = 4.833, *p* = 0.028). On the other hand, knowledge level was not significantly associated with age group (*χ* ^2^ = 3.027, *p* = 0.387), education level (*χ* ^2^ = 1.556, *p* = 0.212), work status (*χ* ^2^ = 0.866, *p* = 0.352), gestational age (*χ* ^2^ = 0.216, *p* = 0.642), parity (*χ* ^2^ = 1.961, *p* = 0.161), or physical activity level (*χ* ^2^ = 0.441, *p* = 0.802).

**TABLE 3:** Association between sociodemographic characteristics and knowledge, attitudes, and practices levels among the study participants.

Variable	Knowledge level				Attitudes levels				Practices levels			
	Good *N* (%)	Poor *N* (%)	*χ* ^2^	*p*	Positive *N* (%)	Negative *N* (%)	*χ* ^2^	*p*	Good *N* (%)	Bad *N* (%)	*χ* ^2^	*p*
Age												
≤ 20	3 (60.0)	2 (40.0)	3.027	0.387	3 (60)	2 (40)	0.727	0.867	3 (60.0)	2 (40.0)	4.491	0.213
21–27	28 (45.2)	34 (54.8)			29 (46.8)	33 (53.2)			27 (43.5)	35 (56.5)		
28–34	25 (58.1)	18 (41.9)			18 (41.9)	25 (58.1)			27 (62.8)	16 (37.2)		
≥ 35	22 (61.1)	14 (38.9)			17 (47.2)	19 (52.8)			16 (44.4)	20 (55.6)		
Education												
Secondary and above	75 (54.7)	62 (45.3)	1.556	0.212	64 (46.7)	73 (53.3)	0.609	0.508	70 (51.1)	67 (48.9)	1.066	0.494
Below secondary	3 (33.3)	6 (66.7)			3 (33.3)	6 (66.7)			3 (33.3)	6 (66.7)		
Work status												
Unemployed	33 (49.3)	34 (50.7)	0.866	0.352	27 (40.3)	40 (59.7)	1.559	0.212	27 (40.3)	40 (59.7)	4.662	0.031*
Employed	45 (57.0)	34 (43.0)			40 (50.6)	39 (49.4)			46 (58.2)	33 (41.8)		
Ethnicity												
Non‐Malay	33 (66.0)	17 (34)	4.833	0.028*	24 (48.0)	26 (52.0)	0.136	0.712	27 (54.0)	23 (46.0)	0.487	0.601
Malay	45 (46.9)	51 (53.1)			43 (44.8)	53 (55.2)			46 (47.9)	50 (52.1)		
Gestation age												
1–12 weeks	2 (66.7)	1 (33.3)	0.216	0.642	2 (66.7)	1 (33.3)	0.532	0.466	2 (66.7)	1 (33.3)	0.340	0.560
> 12 weeks	76 (53.1)	67 (46.9)			65 (45.5)	78 (54.5)			71 (49.7)	72 (50.3)		
Parity												
Nulliparous	40 (59.7)	27 (40.3)	1.961	0.161	30 (44.8)	37 (55.2)	0.062	0.868	37 (46.8)	42 (53.2)	0.690	0.406
Multiparous	38 (48.1)	41 (51.9)			37 (46.8)	42 (53.2)			36 (53.7)	31 (46.3)		
Physical activity												
Sedentary activity	64 (52.9)	57 (47.1)	0.441	0.802	57 (47.1)	64 (59.2)	5.509	0.064	61 (50.4)	60 (49.6)	1.149	0.563
Light activity	10 (52.6)	9 (47.4)			10 (52.6)	9 (47.4)			8 (42.1)	11 (57.9)		
Moderate activity	4 (66.7)	2 (33.3)			0 (0.0)	6 (100.0)			4 (66.7)	2 (33.3)		

*Note:* Sedentary activity (< 1.5 MET); light activity (1.5–3.0 MET); moderate activity (3.0–6.0 MET); vigorous activity (> 6.0 MET). The level of significance and was considered at *p ≤ 0.05.

Furthermore, the association of participants' attitudes toward vitamin D and physical activity level was near significant (*χ* ^2^ = 5.509, *p* = 0.064). However, there was no significant association between attitudes level and age (*χ* ^2^ = 0.727, *p* = 0.867), education (*χ* ^2^ = 0.609, *p* = 0.508), ethnicity (*χ* ^2^ = 0.136, *p* = 0.712), gestational age (*χ* ^2^ = 0.532, *p* = 0.466), parity (*χ* ^2^ = 0.062, *p* = 0.868), and work status (*χ* ^2^ = 1.559, *p* = 0.212).

Participant's practice levels were significantly associated with work status (*χ* ^2^ = 4.662, *p* = 0.031), with employed participants reporting a higher proportion of good practices (58.2%) compared to unemployed participants (40.3%). However, no significant associations were found between practice level and age (*χ* ^2^ = 4.491, *p* = 0.213), education (*χ* ^2^ = 1.066, *p* = 0.494), ethnicity (*χ* ^2^ = 0.487, *p* = 0.601), gestational age (*χ* ^2^ = 0.340, *p* = 0.560), parity (*χ* ^2^ = 0.690, *p* = 0.406), or physical activity (*χ* ^2^ = 1.149, *p* = 0.563).

We performed a binary logistic regression analysis to determine the associations between selected sociodemographic characteristics and the levels of vitamin D knowledge, attitudes, and practices. We found that the participant's ethnicity was significantly associated with knowledge level, which indicated that Malay participants had lower odds of having good knowledge compared to non‐Malay participants (aOR = 0.420, 95% CI: 0.195–0.905, *p* = 0.027). This suggests that non‐Malays were more likely to possess adequate knowledge of vitamin D than their Malay counterparts.

On the other hand, employment status, parity, and gestational age did not show statistically significant associations with knowledge level. There was no significant association between employment status (aOR = 0.877, *p* = 0.736), parity (aOR = 0.593, *p* = 0.160) and gestational age (aOR = 0.464, *p* = 0.539) with knowledge level.

None of the examined sociodemographic variables were significantly associated with attitudes toward vitamin D. Malay ethnicity showed no significant difference in positive attitude compared to non‐Malay women (aOR = 1.014, 95% CI: 0.485–2.122, *p* = 0.970). Similarly, employment (aOR = 1.674, *p* = 0.180), multiparity (aOR = 1.324, *p* = 0.440), and gestational age > 12 weeks (aOR = 0.419, *p* = 0.486) were not significantly associated with attitude levels.

A statistically significant association was found between employment status and vitamin D‐related practices. Employed participants were two times more likely to demonstrate good practices compared to unemployed participants (aOR = 2.036, 95% CI: 1.002–4.139, *p* = 0.049). According to our analysis, participants' ethnicity, parity, and gestational age were not significantly associated with practice levels, as shown in Table 4.

**TABLE 4:** Binary Logistic regression analysis on associations between selected sociodemographic characteristics and knowledge, attitdes and practices levels among the study participants.

Variable	Knowledge level		Attitudes level		Practices level	
	aOR (95% CI)	*p*	aOR (95% CI)	*p*	aOR (95% CI)	*p*
Ethnicity						
Non‐Malay	Reference		Reference		Reference	
Malay	0.420 (0.195–0.905)	0.027*	1.014 (0.485–2.122)	0.970	0.420 (0.461–0.969)	0.934
Work status						
Unemployed	Reference		Reference		Reference	
Employed	0.877 (0.410–1.877)	0.736	1.674 (0.788–3.559)	0.180	2.036 (1.002–4.139)	0.049*
Parity						
Nulliparous	Reference		Reference		Reference	
Multiparous	0.593 (0.286–1.228)	0.160	1.324 (0.649–2.704)	0.440	0.977 (0.479–1.992)	0.950
Gestation age						
1–12 weeks	Reference		Reference		Reference	
> 12 weeks	0.464 (0.040–5.390)	0.539	0.419 (0.036–4.837)	0.486	0.526 (0.044–6.241)	0.611

*Note:* Binary logistic regression; aOR is the adjusted odd ratio at 95% CI. The level of significance and was considered at *p ≤ 0.05.

## 4 Discussion

### 4.1 Knowledge of Vitamin D Among Study Subjects

The present study discovered a good level of knowledge among study participants. This is consistent with the results of previous studies (Aghaei et al. 2021; Aljefree 2017). Although there have been mixed findings regarding the knowledge level of pregnant mothers, other findings are not in agreement with the results of our study (Abdullah et al. 2021; Toher et al. 2014). The differences in the level of knowledge could be attributed to differences in socio‐demographic factors, such as educational attainment, age, and marital status. A similar study conducted in Denmark among Western ethnic minorities reported that vitamin D knowledge level was commendable among young pregnant mothers with higher educational attainment (Özel et al. 2020). Furthermore, similar results were found among pregnant women in the United Kingdom (Connor et al. 2018), Ireland, Asia, Africa, the Middle East, and North African origins (Toher et al. 2014) as well as Southeast Asia (Maryam et al. 2021). The finding of a good knowledge level on vitamin D could be due to the predominance of young participants between 21 and 27 years. Owing to the development of information technology and the generation of the Internet, information on vitamin D can easily be obtained on social media. Moreover, it could be the result of their higher educational attainment and knowledge from their schools, and some of the participants could have been from science‐based backgrounds where they learned the significance of vitamin D nutrients.

### 4.2 Attitudes on Vitamin D Among the Study Participants

As reported in other studies, attitudes related to vitamin D are associated with socio‐cultural practices. The present study found relatively negative attitudes toward vitamin D among the study participants. The results of previous studies were in tandem with our findings (Abdullah et al. 2021; Curtis et al. 2018; Haq et al. 2017). It has been reported that participants' education, occupation, ethnicity, and marital status were influenced by attitudes toward vitamin D when the model of multivariate linear regression was used after taking into account potential compounding variables (aOR = 10.51; −19.59 to 1.42 at 95% CI) (Amegah et al. 2018). However, previous work conducted among female office workers was contrary to our findings, although our study subjects were pregnant women. A similar trend was found among pregnant women in Pakistan (Maryam et al. 2021), the UK (Webb et al. 2016), and African pregnant women in Nigeria (Fasola et al. 2018), Kenya (Cole 2014), and Australia (Vu et al. 2010). However, based on our results, nearly half of the participants 76 (52.1%) believed (disagreed) that urbanization lowers the chance of sunlight exposure. This is not in tandem with the findings of a previous study, which found that 51% of the participants agreed that urbanization could be one of the factors causing the restriction of sunlight exposure (Jamil et al. 2019). This is probably because our subjects were selected from urban areas, and because of the advancement in the standard of living, some city dwellers live in high‐rise buildings with no provision of balconies and recreational areas that allow sunlight exposure. Moreover, other participants had a sedentary lifestyle, which decreased the likelihood of exposure to sunlight. Perhaps our study needs to be substantiated by future studies involving participants from both rural and urban areas.

Furthermore, the present work found a statistically significant relation between subjects' vitamin D status and level of attitudes. Moreover, participants with a deficiency were found to have a negative mindset toward vitamin D. The variation in attitudes related to vitamin D across various studies could be due to differences in attitudes toward sunlight exposure because of an increase in industrialization that limits physical activities during the daytime, thereby discouraging sunlight exposure and covering the body due to religious and cultural teachings and practices. Moreover, the present study reported significantly higher Malay participants, and due to cultural and religious teachings, Malay women tend to cover their bodies with veils, thereby preventing them from sunlight exposure. However, attitudes toward vitamin D are subjective, as some individuals could be keen to adhere to things that can improve their vitamin D levels, whereas other individuals possess negative attitudes toward exposure to sunlight. For example, a study reported that participants were willing to expose only their faces and hands to sunlight only early in the day or late in the afternoon hours to avoid skin burns due to intense sunlight (Tariq et al. 2020). Initiatives to raise awareness of vitamin D's importance are lacking in pregnant women (Aljefree et al. 2017); poor attitudes toward consuming foods high in vitamin D and lack of use of vitamin D‐rich supplements are among the factors that could lead to a negative mindset toward vitamin D with resultant deficiency (Abdullah et al. 2021).

### 4.3 Practice Levels Related to Vitamin D Among the Study Participants

Despite the few studies that assessed vitamin D‐related practice, our study demonstrated relatively inconsistent levels of practice among the participants. This is in line with previous studies conducted in Southeast Asia (Jamil et al. 2019; Woon et al. 2019), Singapore (Divakar et al. 2020), Pakistan (Haq et al. 2017), China (Yun et al. 2015), the Middle East, Saudi Arabia, Jordan (Abdullah et al. 2021), West Africa, Ghana (Amegah et al. 2018), Iran (Tabrizi et al. 2018), and among populations of European, Asian, African, as well as Middle Eastern and North African pregnant mothers (Blebil et al. 2019). Moreover, previous studies conducted in Iran and Pakistan reported poor practice levels with resultant vitamin D deficiency and insufficiency (Nasir et al. 2018; Tabrizi et al. 2018) respectively, as reported in the present study.

In contrast, other authors found contrary results among pregnant women in the Netherlands, with a good level of practice among 87% of subjects (AnnieJudkins 2006). Consequently, the discrepancies in practices on vitamin D intake might be due to the mixed information regarding the “risk versus benefits” of sunlight exposure, which some people believed can cause skin burns or when in excess can cause skin cancer. Another contributing factor is the misconception of vitamin D dietary sources with the notion that, eating a balanced diet food supplies all the required nutrients.

### 4.4 Vitamin D Deficiency Among the Study Participants and Implications of the Study

Vitamin D deficiency is a widespread public health issue, especially among pregnant women who are at higher risk of complications (Fondjo et al. 2021). In this study, 64.4% of participants were found to be vitamin D deficient. This is consistent with other regional studies reporting rates between 23.2% and 90.4% (Akma et al. 2021; Blebil et al. 2019; Bukhary et al. 2016; Lee et al. 2021; Palaniveloo et al. 2020; Woon et al. 2019). Although many participants had high knowledge about vitamin D, this did not translate into positive attitudes or good practices. Vitamin D deficiency in pregnancy is associated with serious health problems, such as gestational diabetes (Alzaim and Wood 2013; Lacroix et al. 2014), hypertensive disorders of pregnancy (Purswani et al. 2017; Ibrahim et al. 2025), cesarean delivery (Aghajafari et al. 2013; Curtis et al. 2018), preterm birth (Dovnik and Mujezinović 2018; van der Pligt et al. 2018), and poor fetal growth (Wei et al. 2013; Yemane et al. 2021), affecting bone development and increasing the risk of long‐term health issues. It is important to stress that maternal vitamin D deficiency is an essential predictor of both the mother's and the child's well‐being across the life course and could play a crucial role in the developmental origin of health and disease (DoHD). Our findings indicate that having knowledge of vitamin D alone is not enough to prevent individuals from vitamin D deficiency, as the majority of participants had negative attitudes and poor practices. This highlights the need for policies on improving not only knowledge but also attitudes and practices related to vitamin D. Additionally, health education programs should address cultural beliefs, encourage safe sun exposure, promote vitamin D‐rich diets, and ensure access to affordable supplements. Prioritizing these preventive measures can reduce the burden of vitamin D deficiency in pregnancy.

### 4.5 Strength and Limitations

Few studies have targeted pregnant women in the study area. This is the first study in Malaysia, evaluating knowledge, attitudes, and practices on vitamin D and its relationship with vitamin D concentration among pregnant women. The study is not devoid of limitations. Firstly, the current study allowed self‐reporting of some responses which are subjected to constraints, including recall bias. However, efforts have been made to ensure that these challenges are significantly tackled by conducting pre‐enrollment interviews with pregnant women for eligibility before they were included in the study. Another potential setback of the study is the lack of adjustment for potential confounding variables such as sun exposure, dietary intake of vitamin D, and BMI. These factors are known to significantly influence vitamin D levels and may also affect participants' knowledge, attitudes, and practices related to vitamin D. The absence of data on these variables could limit the ability to control for their potential effects, which may have introduced bias in the interpretation of the findings. Future research should consider collecting and adjusting for these variables to provide a more comprehensive understanding of the determinants influencing vitamin D‐related behaviors among pregnant women.

This study has certain limitations that should be acknowledged. First, the use of a convenience sampling strategy in a tertiary hospital setting may have introduced sampling bias. Participants were recruited based on their availability and willingness to participate, which may not accurately reflect the broader population of pregnant women in Malaysia. This approach may have disproportionately included individuals with higher health literacy or greater access to healthcare services, thereby limiting the diversity of perspectives captured in the study.

Second, the external validity of the findings is limited due to the hospital‐based recruitment of participants from the Klang Valley region, a predominantly urban settlement. As such, the results may not be generalizable to pregnant women in rural or geographically diverse settings, where cultural practices, healthcare access, and awareness of vitamin D may differ significantly. To enhance the representativeness and applicability of future research, studies should employ probability‐based sampling methods and include participants from a broader range of healthcare settings, including rural and semi‐urban areas across different regions of Malaysia.

## 5 Conclusion

Despite the high level of knowledge about vitamin D among the participants, the majority had negative attitudes, inconsistent practice levels, and a high prevalence of vitamin D deficiency. The findings underscore the need for policies and culturally acceptable strategies such as health education and awareness programs aimed at promoting attitudes and practices to prevent vitamin D deficiency in pregnancy.

## Author Contributions

**Yakubu Ibrahim:** conceptualization (equal), data curation (equal), formal analysis (lead), investigation (lead), methodology (equal), writing – original draft (lead), writing – review and editing (lead). **Amilia Afzan Mohd Jamil:** conceptualization (lead), data curation (lead), formal analysis (equal), methodology (lead), project administration (lead), supervision (lead), writing – review and editing (supporting). **Nurul Iftida Basri:** investigation (supporting), methodology (equal), project administration (supporting), supervision (supporting), writing – review and editing (supporting). **Simran Lau Sher Reen:** data curation (supporting), formal analysis (supporting), methodology (supporting). **Muhammad Daniel Iman Asmunni:** data curation (supporting), formal analysis (supporting), methodology (supporting). **Nirosha Govidarajoo:** data curation (supporting), formal analysis (supporting), methodology (supporting).

## Ethics Statement

The study was approved by the Universiti Putra Malaysia ethics and research committee involving human subjects, approval number: UPM/TNCPI/RMC/1.4.18.2 (JKEUPM). The study was conducted in accordance with the ethical principles of medical research involving human subjects, as enshrined in the declaration of Helsinki (1975), as amended 2004. Informed consent was sought from all the eligible participants before recruitment into the study.

## Conflicts of Interest

The authors declare no conflicts of interest.

## Supporting information


Table S1.

## Data Availability

The data that support the findings of this study are available on request from the corresponding author.
