# Supplementary material for: Vitamin D Knowledge, Attitudes, Practices and Serum Concentration Among Pregnant Women Attending a Malaysian Tertiary Hospital
Source: Food Sci Nutr. 2025 Jul 9;13(7):e70575. doi: 10.1002/fsn3.70575 (PMC12241716; doi:10.1002/fsn3.70575)
Supplement: Supplementary file 1 — Table S1. [file FSN3-13-e70575-s001.docx]

**SUPPLEMENTARY TABLES**

**Table S1: Responses to the vitamin D related knowledge statements among the study participants**

|  | **Knowledge related statements** | **Wrong** | **Not sure** | **Correct** |
| --- | --- | --- | --- | --- |
| 1. | Vitamin D deficiency may be associated with pregnancy complications such as gestational diabetes, hypertensive disorders of pregnancy and preterm delivery. | 12(8.2) | 91(623) | 43(29.3) |
| 2. | Sunlight is one of the key sources of vitamin D synthesis in the body. | 8(5.5) | 109(74.7) | 29(19.9) |
| 3. | Pregnant women with darker skin require a longer period of time to produce vitamin D from sunlight as compared with women with fairer skin. | 52(35.6) | 38(26.0) | 56(38.4) |
| 4. | The only body part that needs to be exposed to sunlight is the face. | 85(58.2) | 29(19.9) | 32(21.9) |
| 5. | In Peninsular Malaysia, the ideal time to be exposed to sunlight is 10 a.m. | 43(29.5) | 67(45.9) | 36(24.7) |
| 6. | Pregnant women have a low risk of vitamin D deficiency. | 74(39.3) | 29(13.6) | 33(15.4) |
| 7. | The use of sunscreens/umbrella can prevent sunlight from penetrating the skin. | 34(15.9) | 68(46.6) | 44(30.1) |
| 8. | Egg yolk (yellow part) is one of the foods containing vitamin D. | 11(7.5) | 83(56.8) | 52(35.6) |
| 9. | Dairy products such as milk, yoghurt, cheese, and butter are enriched with vitamin D. | 17(11.6) | 93(63.7) | 36(24.7) |
| 10. | Cod liver oil is one the example of supplement containing vitamin D. | 15(7.0) | 84(57.5) | 47(32.2) |
| 11. | Vegetables are among the food sources containing vitamin D. | 30(20.5) | 25(58.9) | 30(20.5) |
| 12. | Fatty fish such as mackerel, tuna, and salmon are good vitamins D sources. | 22(15.1) | 89(61.0) | 35(24.0) |

**Table S2: Responses to the vitamin D related attitudes statements among the study participants**

|  | **Attitudes related statements** | **Strongly Disagree** | **Disagree** | **Neutral** | **Agree** | **Strongly Agree** |
| --- | --- | --- | --- | --- | --- | --- |
| 1. | Vitamin D deficiency will lead to serious complications of pregnancy. | 76(52.1) | 17(11.6) | 31(21.2) | 19(13.0) | 3(2.1) |
| 2. | Exposure to sunlight can reduce the risk of pregnancy complications/disorders. | 55(37.7) | 38(26.0) | 33(22.6) | 17(11.6) | 3(2.1) |
| 3. | I still have time and am willing to improve my vitamin D status despite being low. | 21(14.4) | 39(26.7) | 71(48.6) | 15(10.3) | 0(0.0) |
| 4. | Urbanization lowers the chance of being exposed to sunlight. | 17(11.6) | 76(52.1) | 38(26.0) | 15(10.3) | 0(0.0) |
| 5. | While pregnant, we need to expose our bodies to sunlight every day. | 11(7.5) | 50(34.2) | 52(36.6) | 32(21.9) | 1(0.7) |
| 6. | The lack of provision of public recreational parks contributes to a lower chance of sunlight exposure. | 18(12.3) | 26(17.8) | 73(50.0) | 29(19.9) | 0(0.0) |
| 7. | Using umbrella/sun screens becomes necessary every day before going out, even for a short period. | 17(11.6) | 28(19.2) | 75(51.4) | 25(17.1) | 1(0.7) |
| 8. | Avoiding outdoor activities during pregnancy makes pregnant women have lower vitamin D status. | 16(11.0) | 27(18.5) | 64(43.8) | 38(26.0) | 1(0.0) |
| 9. | Pregnant mothers do not need to do housework outside if they have already done some work inside. | 30(20.5) | 39(26.7) | 53(36.3) | 24(16.4) | 0(0.0) |
| 10. | Lack of awareness campaigns on the importance of vitamin D on maternal health leads to poor vitamin D status among pregnant mothers. | 34(23.3) | 37(25.3) | 49(33.6) | 25(17.1) | 1(0.7) |
| 11. | Vitamin D supplementation for pregnant mothers becomes necessary if dietary provision and sunlight exposure are low. | 19(13.0) | 23(15.8) | 52(35.6) | 50(34.2) | 2(1.4) |
| 12. | Vitamin D-enriched foods such as fatty fish, milk and milk products (yoghurts, butter and cheese) are expensive. | 27(18.5) | 34(23.3) | 59(40.4) | 25(17.1) | 1(0.7) |

**Table S3: Responses to the vitamin D related practices statements among the study participants**

|  | **Practices related statements** | **Never** | **Rarely** | **Sometimes** | **Often** | **Always** |
| --- | --- | --- | --- | --- | --- | --- |
| 1. | While I am pregnant, I always do some domestic work outside of my house or workplace. | 40(27.4) | 34(23.3) | 48(32.9) | 22(15.1) | 2(1.4) |
| 2. | While pregnant, I used an umbrella or hat every time of the day when under the sun. | 44(30.1) | 43(29.5) | 49(33.6) | 10(6.8) | 0(0.0) |
| 3. | While pregnant, I use sunscreen on my face and hands while out of doors for more than 1 hour. | 54(37.0) | 29(19.9) | 45(30.8) | 17(11.6) | 1(0.5) |
| 4. | While pregnant, I do walk outside under the sun every time in day. | 37(25.3) | 33(22.6) | 59(40.4) | 16(11.0) | 1(0.7) |
| 5. | I only expose my body to sunlight via windows because of the nature of my house or office (no space for sun exposure). | 27(18.5) | 39(26.7) | 55(37.7) | 22(15.1) | 6(4.1) |
| 6. | I always cover my head and body with a scarf and long clothing outside or working under the sun. | 30(20.5) | 35(24.0) | 53(36.3) | 22(15.1) | 6(4.1) |
| 7. | Due to Malaysia’s humid and hot weather, I am most comfortable working indoors in my house or office. | 17(11.6) | 23(15.8) | 62(42.5) | 38(26.0) | 6(4.1) |
| 8. | While pregnant, I take fish oil or fatty fish (salmon, tuna, sardine, and mackerel) at least two servings per day. | 40(27.4) | 29(19.9) | 57(39.0) | 16(11.0) | 4(2.7) |
| 9. | When eating an egg, I only eat the white part. | 54(37.0) | 38(26.0) | 45(30.8) | 8(5.5) | 1(0.7) |
| 10. | While pregnant, I always take dairy products (milk, yoghurt, butter, and cheese) with no more than two servings per day. | 40(24.4) | 22(15.1) | 56(38.4) | 22(15.1) | 6(4.1) |
| 11. | I am always keen on reading food labels for nutrition information before buying and always choose vitamin enriched-enriched foods. | 27(18.5) | 29(19.9) | 62(42.5) | 24(16.4) | 4(2.7) |
| 12. | While I am pregnant, I always take vitamin D supplements in addition to other supplements. | 43(29.5) | 27(18.5) | 52(35.6) | 21(14.4) | 3(2.1) |

**Table S4: Responses to pregnancy physical activity questions among the study participants**

|  | **Pregnancy Physical Activity Questions** | **None** | **Less than ½ hour per day** | **½ to almost 1 hour per day** | **1 to almost 2 hours per day** | **2 to almost 3 hours per day** | **3 or more hours per day** |
| --- | --- | --- | --- | --- | --- | --- | --- |
| 1. | Preparing meals (cook, set the table, wash dishes) | 11 (7.7%) | 16(11.2%) | 45(31.5%) | 38 (26.6%) | 21(14.7%) | 12 (8.4%) |
| 2. | Carrying children. | 84(57.5%) | 11 (7.5%) | 15 (10.3%) | 12 (8.2%) | 11 (7.5%) | 13 (8.9%) |
| 3. | Sitting and reading, talking, or on the phone while not at work. | 6 (4.1%) | 14 (9.6%) | 26 (17.8%) | 25 (17.1%) | 21 (14.4%) | 54 (37.0%) |
| 4 | Shopping (for food, clothes or other items) | 4 (2.7%) | 34 (23.3%) | 52 (35.6%) | 34 (23.3%) | 12 (8.2%) | 10 (6.8%) |
| 5 | Light cleaning (make beds, laundry, iron) | 4 (2.7%) | 34 (23.3%) | 52 (35.6%) | 34 (23.3%) | 12 (8.2%) | 10 (6.8%) |
| 6 | Heavier cleaning (vacuum, mop, sweep, wash windows) | 15(10.3%) | 42 (28.8%) | 45 (30.8%) | 31 (21.2%) | 9 (6.2%) | 4 (2.7%) |
| 7 | Mowing the lawn while on a riding mower | 113(77.4%) | 17(11.6%) | 9(6.2%) | 3(2.1%) | 2(1.4%) | 2(1.4%) |
| 8 | Watching TV or a video | 12 (8.2%) | 18(12.3%) | 32(21.9%) | 20(13.7%) | 39(26.7%) | 25(17.1%) |
| 9 | Strolling for fun or exercise | 10(6.8%) | 47(32.2%) | 36(24.7%) | 31(21.2%) | 14(9.6%) | 8(5.5%) |
| 10 | Walking more quickly for fun or exercise | 32(21.9%) | 39(26.7%) | 41(28.1%) | 22(15.1%) | 7(4.8%) | 5(3.4%) |
| 11 | Walking quickly up hills for fun or exercise | 66(45.2%) | 35(24.0%) | 23(15.8%) | 13(8.9%) | 5(3.4%) | 4(2.7%) |
| 12 | Jogging | 70(47.9%) | 43(29.5%) | 15(10.3%) | 13(8.9%) | 3(2.1%) | 2(1.4%) |
| 13 | Dancing | 88(60.3%) | 28(19.2%) | 14(9.6%) | 13(8.9%) | 0 (0%) | 3(2.1%) |
| 14 | Prenatal exercise class | 91(62.3%) | 24(16.4%) | 17(11.6%) | 8(5.5%) | 3(2.1%) | 3(2.1%) |
| 15 | Swimming | 113(77.4%) | 17(11.6%) | 9(6.2%) | 3(2.1%) | 2(1.4%) | 2(1.4%) |
| 16 | Sitting at work or in class | 16(11.0%) | 12(8.2%) | 37(25.3%) | 18(12.3%) | 17(11.6%) | 46(31.5%) |
| 17 | Walking quickly at work while carrying things (heavier than a 4 litres jug of milk) | 63(43.2%) | 40(27.4%) | 20(13.7%) | 15(10.3%) | 4(2.7%) | 4(2.7%) |
| 18 | Standing or slowly walking at work while carrying things (heavier than a 4 litres jug of milk) | 63(43.2%) | 40(27.4%) | 20(13.7%) | 15(10.3%) | 4(2.7%) | 4(2.7%) |
| 19 | Walking quickly at work, not carrying anything | 65(44.5%) | 37(25.3%) | 25(17.1%) | 9(6.2%) | 4(2.7%) | 6(4.1%) |
| 20 | Standing or slowly walking at work, not carrying anything | 37(25.3%) | 33(22.6%) | 42(28.8%) | 11(7.5%) | 12(8.2%) | 11(7.5%) |
